# Supplementary figures and images for: Dynamical Task Switching in Cellular Computers
Source: Life (Basel). 2019 Jan 26;9(1):14. doi: 10.3390/life9010014 (PMC6463194; doi:10.3390/life9010014)

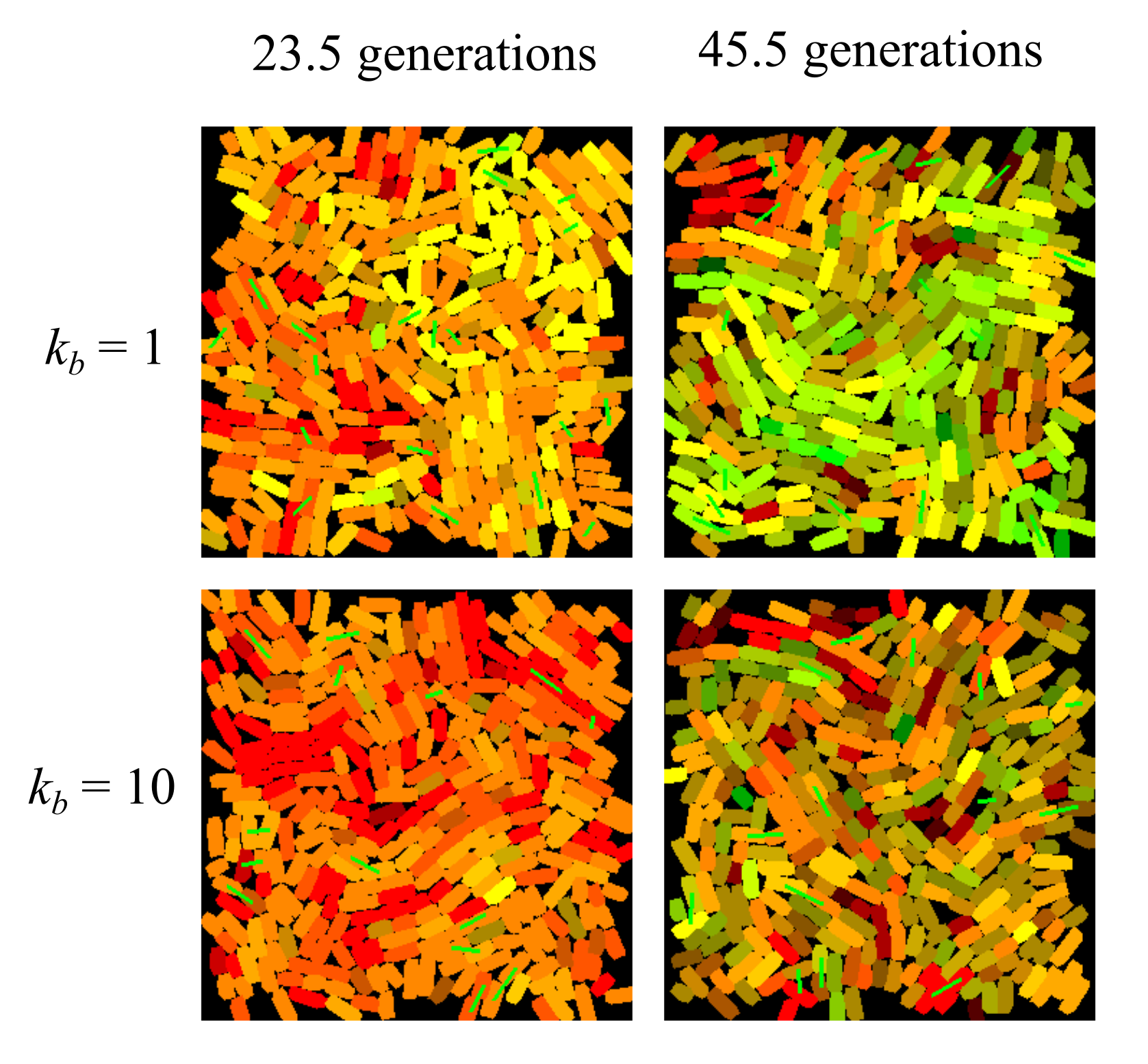

Supplement: Supplementary file 1 [file life-09-00014-s001.zip › Figure_S1.tiff]
